# On the Sparse Beamformer Design

**DOI:** 10.3390/s18103536

**Published:** 2018-10-19

**Authors:** Mingjie Gao, Ka Fai Cedric Yiu, Sven Nordholm

**Affiliations:** 1 School of Mathematical Sciences, South China Normal University, Guangzhou 510000 , China; mjgao@m.scnu.edu.cn; 2 Department of Applied Mathematics, The Hong Kong Polytechnic University, Hunghom, Kowloon, Hong Kong, China; 3 Department of Electrical and Computer Engineering, Curtin University, Perth 6102, Australia; s.nordholm@curtin.edu.au

**Keywords:** beamformer design, signal enhancement, array pruning, sparse filters

## Abstract

In designing acoustic broadband beamformers, the complexity can grow significantly when the number of microphones and the filter length increase. It is advantageous if many of the filter coefficients are zeroes so that the implementation can be executed with less computation. Moreover, the size of the array can also be pruned to reduce complexity. These problems are addressed in this paper. A suitable optimization model is proposed. Both array pruning and filter thinning can be solved together as a two-stage optimization problem to yield the final sparse designs. Numerical results show that the complexity of the designed beamformers can be reduced significantly with minimal effect on performance.

## 1. Introduction

Beamforming is a spatial filtering technique used to enhance the required signal via a sensor array for directional signal transmission or reception [1,2]. With a fixed configuration, the steering vectors of the desired signals can be estimated together with their directions-of-arrival [3]. More elaborated physical signal propagation models have also be employed to describe complicated wave phenomena [4]. As a result, the beamformer design problem can be formulated as an optimization problem similar to the design of multidimensional digital filters. Various optimization methods, such as linear programming techniques [5], quadratic programming techniques [6], and second-order cone programming [7] have been applied. If the beamformers are applied in the near-field of the speaker, it becomes a broadband design problem and several optimization methods have been developed. These include the use of quadratic programming [8], multicriteria formulation [9], linear programming [10], and semi-definite programming [11,12]. We have also analytically investigated the performance limit of the optimization when the filter length is long and the number of microphones is large [13]. As the dimension of the beamformer increases, the optimization problems become large-scale and are difficult to handle, even with state-of-the-art optimization software. Additionally, the complexity of the beamforming system becomes very high.

In designing beamformers, it is advantageous to have filters with many zeroes. In this way, the implementation complexity can be reduced significantly. Therefore, the design of sparse beamforming filters is of great interest. In solving this $l_{0}$-norm problem, an often subproblem is to employ a $l_{1}$-norm as a linear relaxation of the original problem, and iterate on the number of zeroes via a successive thinning technique [14,15]. When the $l_{1}$-norm is employed, the problem becomes a non-strictly convex semi-infinite programming problem. For fast convergence and at the same time to reduce the magnitudes of unnecessary filter coefficients, a perturbation exchange algorithm is proposed. The idea is to add a small perturbation to the objective function, making the problem strictly convex. Under the influence of a small perturbation to the objective function, we obtain similar frequency response to the traditional method [10]. Moreover, an efficient exchange method was proposed for a convex semi-infinite programming problem in [16] which has low complexity. During the exchange process, global optima of the exchange points are not required for addition, but only those points with constraint violations. In dropping active points, the Lagrange multipliers are used to remove all inactive constraints. The new add–drop rule saves much computational time. This is particularly important for higher-dimensional design problems after discretization with a huge dimension in the constraint set.

In addition to the sparseness in the filters, it is also important to prune unnecessary microphones in order to reduce the size of the array without affecting the performance very much. Indeed, in the beamforming design problem, the array response changes significantly when the microphone positions change [17]. This problem has been addressed partially in the array thinning technique [18,19], which is essentially one-dimensional with application mainly in antenna design. A multi-dimensional array configuration design problem has been proposed in [20,21] which allows the microphone positions to be optimized in the multi-dimensional spatial domain. As an extension, in order to prune unnecessary microphones, it is possible to formulate the sparse configuration design problem by adding a penalty term to the configuration design problem in order to drive redundant microphones together for successive pruning.

In this paper, we formulate and propose a novel algorithm for the sequential pruning of a microphone array, and at the same time tackle the sparse design of beamforming filters through successive thinning. We formulate the optimization problem based on the minimax approach, although the method should work equally well for the least-squares or the minimum mean output energy approaches [22,23]. Numerical results have shown that it is possible to have a region of approximately equal performance when the sparsity increases, in both filters and microphones. Therefore, the proposed methods can indeed reduce the complexity of the beamformer array and filters significantly without performance degradation. In the following sections, the beamformer design problem is first described in Section 2. Then, the formulation of the microphone array pruning problem is presented in Section 3 together with the perturbation exchange algorithm. Finally, numerical results are given in Section 4.

## 2. Beamformer Design Formulation

In a typical environment, a beamformer contains a series of microphones placed in pre-defined locations. Behind each microphone, there is an FIR filter attached for processing the received sound signals [9]. However, in the beamforming design problem, the array response may change significantly when the microphone positions change. Thus, we should not ignore the configuration of the microphone array. Let the beamformer have *N* microphones and let each FIR filter have *L* taps. Denote the position vector of the source signal by s and the position vector of the *i*-th microphone by $r_{i}$. Let $\gamma =(r_{1},r_{2},\cdots ,r_{N})$, and the region of all possible locations is given by $\Gamma^{N}=\Gamma \times \cdots \times \Gamma$.

The transfer function from the source to the *i*-th microphone is given by

(1) $$A_{i}\left(s,f,\gamma \right)=\frac{1}{∥s-\gamma_{i}∥}e^{-j2\pi f∥s-\gamma_{i}∥/c}.$$

The array response is therefore given by

(2) $$a\left(s,f,\gamma \right)={\left(A_{1}\left(s,f,\gamma \right),\ldots ,A_{N}\left(s,f,\gamma \right)\right)}^{⊺},$$

and the beam response is

$$G\left(s,f,\gamma \right)=w^{⊺}d\left(s,f,\gamma \right)$$

with

$$d\left(s,f,\gamma \right)=a\left(s,f,\gamma \right)⊗d_{0}\left(f\right),$$

where ⊗ is the Kronecker product, and $d_{0}\left(f\right)={\left(1,e^{-j2\pi f/f_{s}},\ldots ,e^{-j2\pi f\left(L-1\right)/f_{s}}\right)}^{⊺}$ is the filter response vector. For a given array configuration, this beamforming design problem can be formulated as a minimax problem:

(3) $$\min_{w\in R^{NL},\gamma \in \Gamma^{N}}\max_{(s,f)\in \Omega }\left|w^{⊺}d\left(s,f,\gamma \right)-G_{d}\left(s,f,\gamma \right)\right|,$$

where $G_{d}\left(s,f,\gamma \right)$ is the specified desired response of the broadband beamformer.

Following [10], we expand the complex functions as

$$d\left(s,f,\gamma \right)=d_{1}\left(s,f,\gamma \right)+jd_{2}\left(s,f,\gamma \right),$$

$$G_{d}\left(s,f,\gamma \right)=G_{d_{1}}\left(s,f,\gamma \right)+jG_{d_{2}}\left(s,f,\gamma \right),$$

and denote

$$u\left(s,f,\gamma \right)=(w^{⊺}d_{1}\left(s,f,\gamma \right)-G_{d_{1}}\left(s,f,\gamma \right)),$$

$$v\left(s,f,\gamma \right)=(w^{⊺}d_{2}\left(s,f,\gamma \right)-G_{d_{2}}\left(s,f,\gamma \right)).$$

By introducing a slack variable δ

$$\delta =\max_{(s,f)\in \Omega }\left|u\left(s,f,\gamma \right)+jv\left(s,f,\gamma \right)\right|,$$

the above minimax problem can be further written as

(4) $$\left\{\begin{array}{cc} \min_{w\in R^{NL},\gamma \in \Gamma^{N},\delta }\; & \delta \\ \mathrm{s.t.}\; & |u(s,f,\gamma )+jv(s,f,\gamma )|\leq \delta \;\;\;\;\;\forall (s,f)\in \Omega . \end{array}\right.$$

We can actually control the real part and the imaginary part separately. Using the $l_{1}$ norm as a linear relaxation, and introducing two new variables as

$$z_{1}=\max_{(s,f)\in \Omega }|u\left(s,f,\gamma \right)|,\;\;\;z_{2}=\max_{(s,f)\in \Omega }\left|v\left(s,f,\gamma \right)\right|,$$

we convert the above problem into the following problem:

(5) $$\left\{\begin{array}{cc} \min_{w\in R^{NL},\gamma \in \Gamma^{N},z_{1},z_{2}}\; & \;z_{1}+z_{2}\\ s.t.\; & \left|u\left(s,f,\gamma \right)\right|\leq z_{1}\;\;\;\;\;\forall \left(s,f\right)\in \Omega \\ \; & \left|v\left(s,f,\gamma \right)\right|\leq z_{2}\;\;\;\;\;\forall \left(s,f\right)\in \Omega \end{array}\right.$$

which is equivalent to

(6) $$\left\{\begin{array}{ccc} \min_{w\in R^{NL},\gamma \in \Gamma^{N},z_{1},z_{2}}\; & z_{1}+z_{2}\\ s.t.\; & w^{⊺}d_{1}\left(s,f,\gamma \right)-G_{d_{1}}\left(s,f,\gamma \right)\leq z_{1}\;\;\;\;\;\;\;\;\; & \forall (s,f)\in \Omega \\ \; & -w^{⊺}d_{1}\left(s,f,\gamma \right)+G_{d_{1}}\left(s,f,\gamma \right)\leq z_{1}\;\;\;\;\; & \forall (s,f)\in \Omega \\ \; & w^{⊺}d_{2}\left(s,f,\gamma \right)-G_{d_{2}}\left(s,f,\gamma \right)\leq z_{2}\;\;\;\;\;\;\;\;\; & \forall (s,f)\in \Omega \\ \; & -w^{⊺}d_{2}\left(s,f,\gamma \right)+G_{d_{2}}\left(s,f,\gamma \right)\leq z_{2}\;\;\;\;\; & \forall (s,f)\in \Omega . \end{array}\right.$$

To summarize, the design problem can be formulated as the following semi-infinite programming problem:

(7) $$\left\{\begin{array}{ccc} \min_{z\in R^{NL+2},\gamma \in \Gamma^{N}}\; & b^{T}z\\ s.t.\; & H(s,f,\gamma )z-G(s,f,\gamma )\leq 0\;\;\;\;\; & \forall (s,f)\in \Omega , \end{array}\right.$$

where $z={\left(w,z_{1},z_{2}\right)}^{T}$, $b={\left(0,1,1\right)}^{T}$,

$$H\left(r,f,\gamma \right)=\left(\begin{array}{ccc} d_{1}{\left(s,f,\gamma \right)}^{T} & -1 & 0\\ -d_{1}{\left(s,f,\gamma \right)}^{T} & -1 & 0\\ d_{2}{\left(s,f,\gamma \right)}^{T} & 0 & -1\\ -d_{2}{\left(s,f,\gamma \right)}^{T} & 0 & -1 \end{array}\right),\;G\left(r,f,\gamma \right)=\left(\begin{array}{c} G_{d_{1}}\left(s,f,\gamma \right)\\ -G_{d_{1}}\left(s,f,\gamma \right)\\ G_{d_{2}}\left(s,f,\gamma \right)\\ -G_{d_{2}}\left(s,f,\gamma \right) \end{array}\right).$$

Define

g(z,(s,f,γ))=H(s,f,γ)z-G(s,f,γ).

Then the above problem (2) can be represented by

(8) $$\left\{\begin{array}{ccc} \min_{z\in R^{NL+2},\gamma \in \Gamma^{N}}\; & b^{T}z\\ s.t.\; & g(z,(s,f,\gamma ))\leq 0\;\;\;\;\; & \forall (s,f)\in \Omega . \end{array}\right.$$

## 3. Microphone Array Pruning Formulation

For microphone array configuration design, we formulate the problem as

(9) $$\min_{\gamma \in \Gamma^{N}}b^{T}z\left(\gamma \right),$$

where z(γ) is the optimal value of the beamformer filter design problem

$$\left\{\begin{array}{ccc} \min_{z\in R^{NL+2}}\; & b^{T}z\left(\gamma \right)\\ s.t.\; & g(z,(s,f,\gamma ))\leq 0\;\;\;\;\; & \forall (s,f)\in \Omega . \end{array}\right.$$

However, if the number of microphones is very large, it will increase the implementation complexity significantly, but not necessarily improve the performance. Thus, we should find a way to prune unnecessary microphones in order to reduce the size of the microphone array without substantially affecting the performance. Let the initial number of microphones be *N*. A penalty term can be added to (9) in order to drive redundant microphones together for subsequent pruning. The final sparse configuration design problem can be formulated as

$$\min_{\gamma \in \Gamma^{N}}b^{T}z\left(\gamma \right)+\delta \sum_{i,j}{∥\gamma_{i}-\gamma_{j}∥}_{2}^{2},$$

where z(γ) is the optimal value of the beamformer filter design problem

$$\left\{\begin{array}{ccc} \min_{z\in R^{NL+2}}\; & b^{T}z\left(\gamma \right)+\epsilon {∥z∥}_{2}^{2}\\ s.t.\; & g(z,(s,f,\gamma ))\leq 0\;\;\;\;\; & \forall (s,f)\in \Omega . \end{array}\right.$$

In the above optimization problem, the added penalty function $\delta \sum_{i,j}{∥\gamma_{i}-\gamma_{j}∥}_{2}^{2}$ in the objective function plays the role of driving redundant microphones together. Clearly, when we increase δ, the redundant microphones will move closer and faster. After solving the above optimization problem, for every pair of microphones, we can calculate the Euclidean distances $d_{ij}={∥\gamma_{i}-\gamma_{j}∥}_{2}$ between every pair of microphones $\gamma_{i}$ and $\gamma_{j}$. Denote the smallest distance by $d_{ab}$. Using $d_{ab}$, we prune the microphone $\gamma_{a}$, so the number of microphones is reduced to N-1. We in turn solve the above optimization problem using N-1 microphones with the designed configuration. We prune one microphone in each iteration until the stopband ripple has increased unacceptably. The final algorithm can be summarized as follows:

Step 1: Set k=0 and solve the following two-stage optimization problem:
  $$\min_{\gamma \in \Gamma^{N-k}}b^{T}z\left(\gamma \right)+\delta \sum_{i,j}{∥\gamma_{i}-\gamma_{j}∥}_{2}^{2},$$
  where z(γ) is the optimal value of the beamformer filter design problem with a small perturbation
  $$\left\{\begin{array}{ccc} \min_{z\in R^{(N-k)L+2}}\; & b^{T}z\left(\gamma \right)+\epsilon {∥z∥}_{2}^{2}\\ s.t.\; & g(z,(r,f,\gamma ))\leq 0\;\;\;\;\; & \forall (r,f)\in \Omega . \end{array}\right.$$
  If stopband ripple increases significantly, stop.
Step 2: The microphones *a* and *b* are chosen to correspond to the smallest Euclidean distance
  $$d_{ab}=\min{∥\gamma_{i}-\gamma_{j}∥}_{2},i,j=1,\cdots ,N-k.$$
Step 3: Prune the microphone $\gamma_{a}$, set k=k+1, and return to Step 1.

In solving the sub-problem (10), discretization is employed to convert the solution space into a finite set of points. Note that we also add ${\epsilon ∥z∥}_{2}^{2}$ in the objective function of the beamformer filter design subproblem so that the objective function becomes strictly convex, which enables the use of modified exchange method [16,24] with a rather low complexity, even for the multi-dimensional design problem. When ϵ is sufficiently small, the approximate solution of the perturbed problem will be very close to the true solution.

Assume that there exists a finite subset $\Omega_{0}$ such that $b^{T}z$ is level bounded on the feasible set, that is, for every a∈R, the set

$$L_{a}^{0}:=\{z\in R^{n};b^{T}z\leq a\;\mathrm{and}\;g\left(z,\left(s,f,\gamma \right)\leq 0\;\mathrm{for}\;\mathrm{all}\;\left(s,f\right)\in \Omega_{0}\right\}$$

is bounded when it is nonempty. For a given finite set $R=\{\left(s_{j},f_{j}\right),j=1,\cdots ,m\}\subset \Omega$, we consider the finite problem denoted by ($BP_{\epsilon }\left(R^{\left(i\right)}\right)$):

(10) $$\left\{\begin{array}{cc} \min_{z\in R^{(N-k)L+2}}\; & b^{T}z\left(\gamma \right)+\epsilon {∥z\left(\gamma \right)∥}_{2}^{2}\\ s.t.\; & g(z,\left(s_{j},f_{j},\gamma \right))\leq 0\;\;\;\mathrm{for}\;\mathrm{any}\;j=1,\cdots ,m \end{array}\right.$$

We use a modified exchange algorithm [24] for this subproblem. The algorithm can be summarized as follows:

Step 0: Choose a finite reference set $R^{\left(0\right)}=\left\{\left(s_{j}^{\left(0\right)},f_{j}^{\left(0\right)}\right),j=1,\cdots ,m_{0}\right\}\subset \Omega$ such that $\Omega_{0}\subset R^{0}$. Let $z^{\left(0\right)}\left(\gamma \right)$ be an optimal solution to $\left(BP_{\epsilon }\left(R^{\left(0\right)}\right)\right)$ and let $\{\lambda (s_{j}^{\left(0\right)},$$f_{j}^{\left(0\right)}),j=1,\cdots ,m_{0}\}\in R^{m}$ be the set of associated multipliers. Set k=0.
Step 1: Find a set $\{\left(s_{new}^{\left(k\right)},f_{new}^{\left(k\right)}\right),new=1,\cdots ,n\}\subset \Omega$ such that
  $$g(z^{\left(k\right)},\left(s_{new}^{\left(k\right)},f_{new}^{\left(k\right)},\gamma \right))>\eta .$$
  If such a point does not exist, then stop. Otherwise, put ${\bar{R}}^{\left(k+1\right)}=R^{\left(k\right)}\cup \left\{\left(s_{new}^{\left(k\right)},f_{new}^{\left(k\right)}\right)\right\}$.
Step 2: Let $z^{\left(k+1\right)}\left(\gamma \right)$ be an optimal solution to $\left(BP_{\epsilon }\left({\bar{R}}^{\left(k+1\right)}\right)\right)$ and let $\left\{\lambda^{\left(k+1\right)}\left(s,f\right),\left(s,f\right)\in {\bar{R}}^{\left(k+1\right)}\right\}$ be the set of associated multipliers.
Step 3: Let
  $$R^{\left(k+1\right)}:\;=\left\{\left(s,f\right)\in {\bar{R}}^{\left(k+1\right)};\left(s,f\right)\in \Omega_{0}\;\;\mathrm{or}\;\;\lambda^{\left(k+1\right)}\left(s,f\right)>0\right\}.$$
  Set k=k+1, and return to Step 1.

One important point in Step 1 is that we choose multiple points satisfying the adding rule instead of just choosing one. This will reduce the number of iterations and shorten the computational time. Furthermore, convergence of the algorithm can be found in [24].

Using the final pruned array, we can then perform the sparse filter design problem. The overall sparse design problem can be tackled in two stages. In the first stage, the microphone array is first pruned. Then, we can initiate stage two of the method to design the sparse beamforming filters using the algorithm presented in [24].

## 4. Numerical Examples

**Figure 1:**
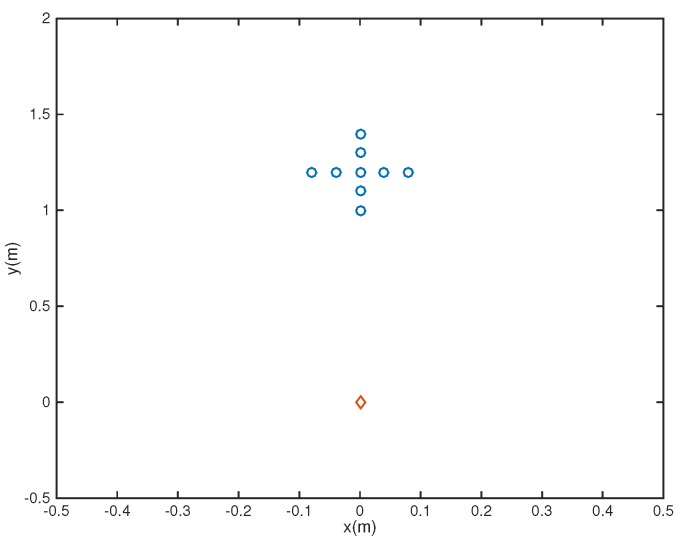
Initial array configuration (Ex1).

In this section, we provide examples to demonstrate the performance of the algorithm which was implemented in MATLAB. We chose the desired response function as

$$\begin{array}{c} G_{d}\left(r,f\right)=\left\{\begin{array}{cc} e^{-j2\pi f\left(\frac{||r-r_{c}\left|\right|}{c}+\frac{L-1}{2}T\right)}, & \;\mathrm{if}\;(r,f)\;\mathrm{is}\;\mathrm{in}\;\mathrm{passband}\;\mathrm{region},\\ 0, & \;\mathrm{if}\;(r,f)\;\mathrm{is}\;\mathrm{in}\;\mathrm{stopband}\;\mathrm{region}, \end{array}\right. \end{array}$$

where $r_{c}$ is the reference central microphone location. In this example, we considered the microphone array pruning problem combined with the design of sparse filters. Assume first that there are nine microphones located at the coordinates {(−0.08, 1.2), (−0.04,1.2), (0,1.2), (0.04,1.2), (0.08,1.2), (0,1.4), (0,1.3), (0,1.1), (0,1)} (Figure 1), with a 26-tap FIR filter behind each element. Note that in the Figures, the speaker is represented by a red diamond while microphones are represented by blue circles. The passband region is defined as

{(x,f):-0.4m≤x≤0.4m,0.5kHz≤f≤1.5kHz},

while the stopband region is simplified as the union of

{(x,f):1.8m≤|x|≤3m,0.5kHz≤f≤1.5kHz},

{(x,f):-3m≤|x|≤3m,2kHz≤f≤4,kHz}.

**Figure 2:**
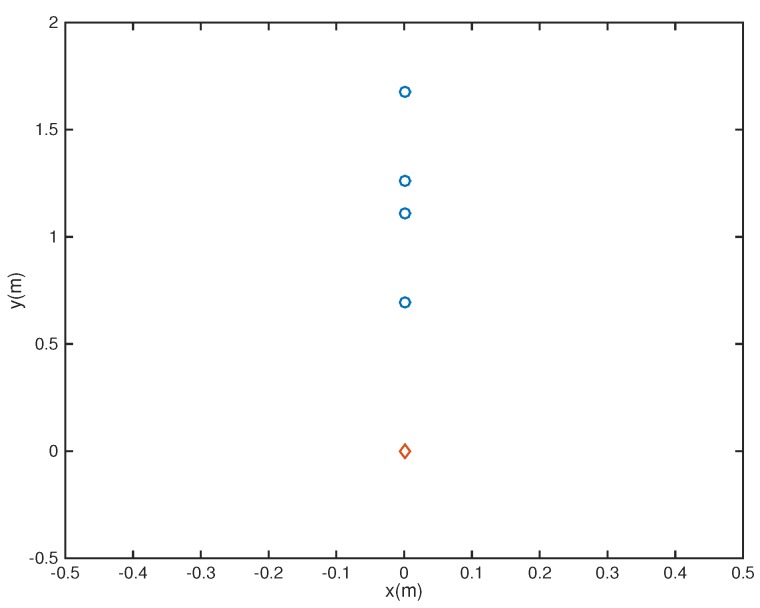
Final array configuration (Ex1).

**Figure 3:**
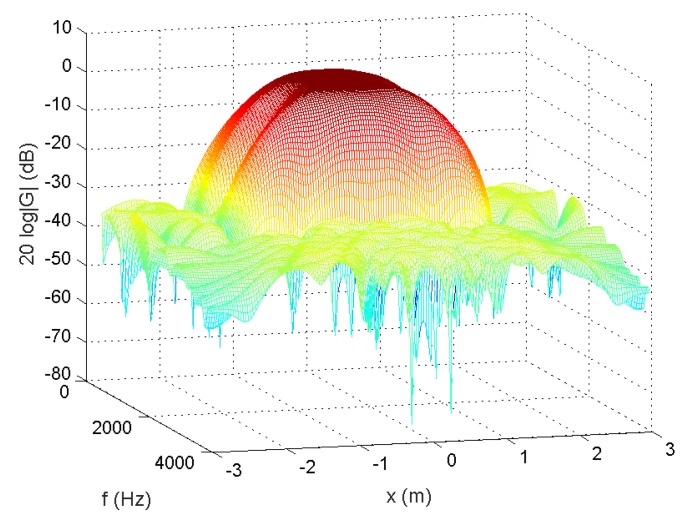
Amplitude of G(r,f), 55.56% of zeroes for microphone arrays (Ex1).

**Figure 4:**
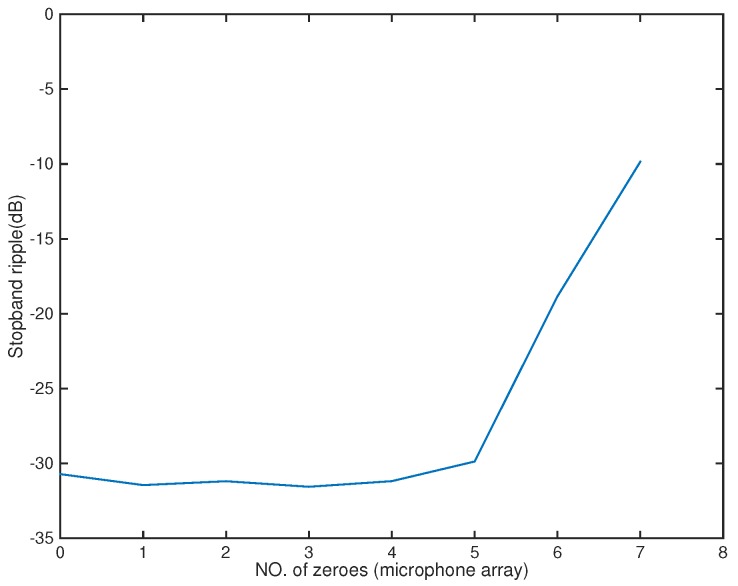
Stopband ripple for microphone arrays (Ex1).

**Figure 5:**
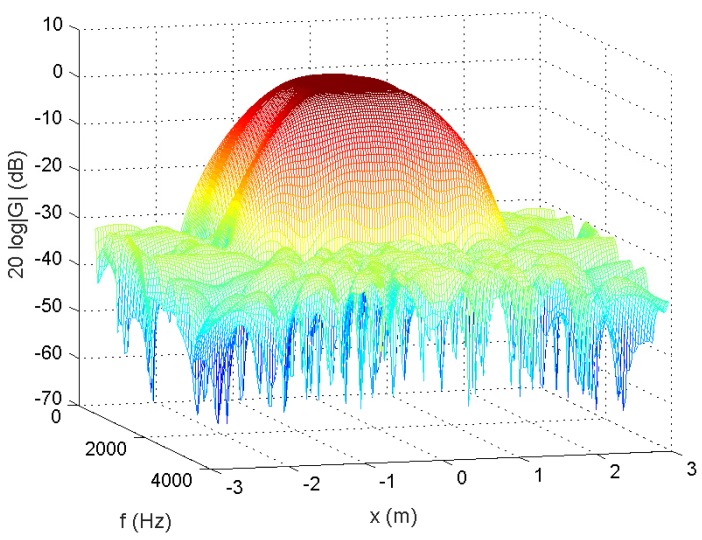
Amplitude of G(r,f), 38.46% of zeroes for filters (Ex1).

**Figure 6:**
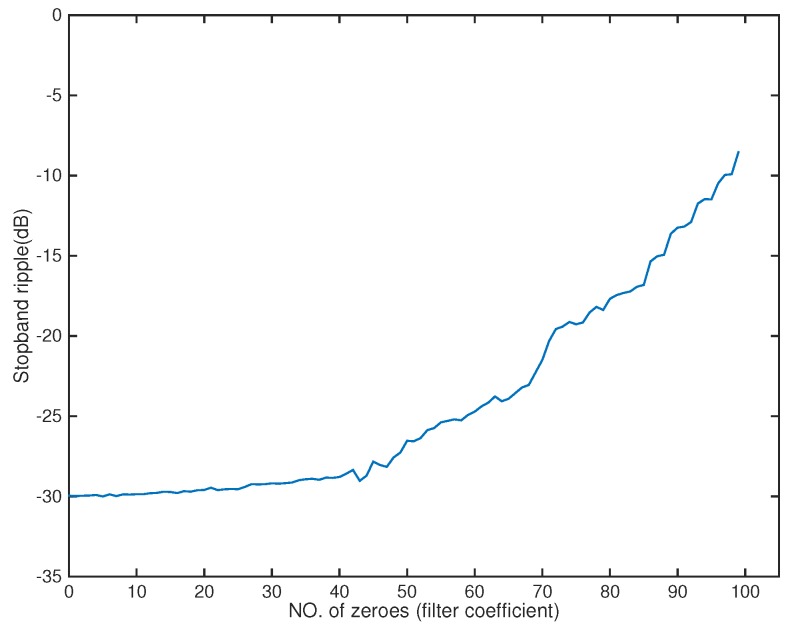
Stopband ripple for filters (Ex1).

In the following, we used the MATLAB functions *fminsearch* and quadprog to solve the microphone pruning problem. For the performance, since stopband ripple is an important indicator of noise reduction, we employed it to demonstrate the effect of sparsity in the designs. After solving the problem, the number of microphones could be decreased to 4 (Figure 2). The amplitude of the actual response G(r,f) is shown in Figure 3, in which the passband is represented by red color. From Figure 4, we can see that the designed beamformer size could be reduced significantly. Using the final pruned array, we could then perform the sparse filter design problem. The amplitude of the actual response G(r,f) is shown in Figure 5. It is observed in Figure 6 that the performance was not affected greatly even when the sparseness increased to about 38% of zero elements for filter coefficient.

**Figure 7:**
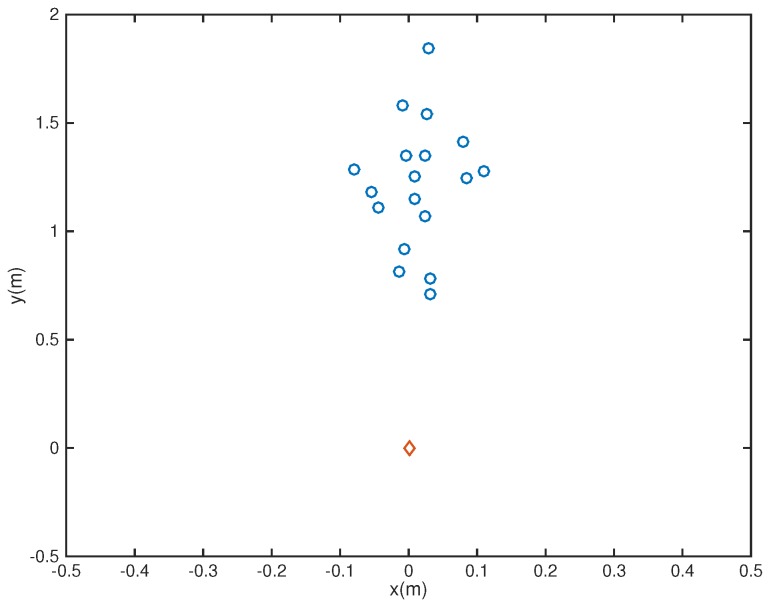
Initial array configuration (Ex2).

In the next example, we considered the microphone array pruning problem using a randomly generated microphone array (Figure 7). The passband region was defined as

{(x,f):-0.4m≤x≤0.4m,0.5kHz≤f≤1.5kHz},

while the stopband region was the union of several parts, as

{(x,f):-0.4m≤x≤0.4m,2kHz≤f≤4kHz},

{(x,f):1.8m≤|x|≤3m,0.5kHz≤f≤1.5kHz},

{(x,f):1.8m≤|x|≤3m,2kHz≤f≤4kHz}.

**Figure 8:**
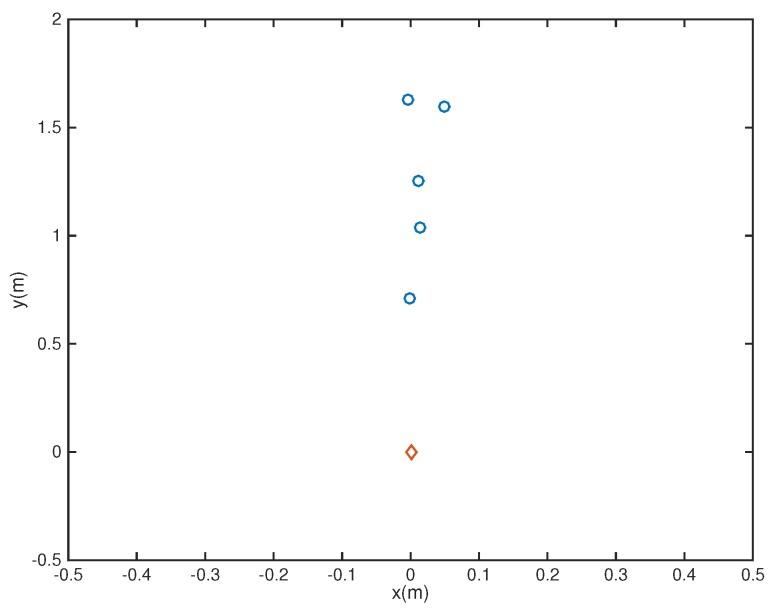
Final array configuration (Ex2).

**Figure 9:**
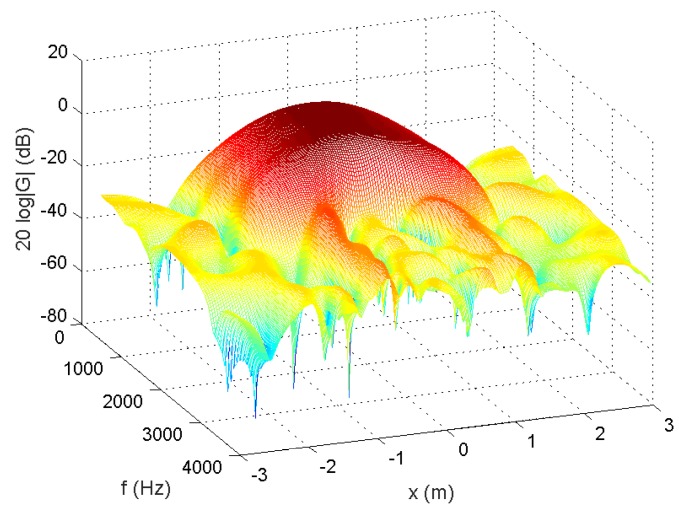
Amplitude of G(r,f), 75% of zeroes for microphone arrays (Ex2).

**Figure 10:**
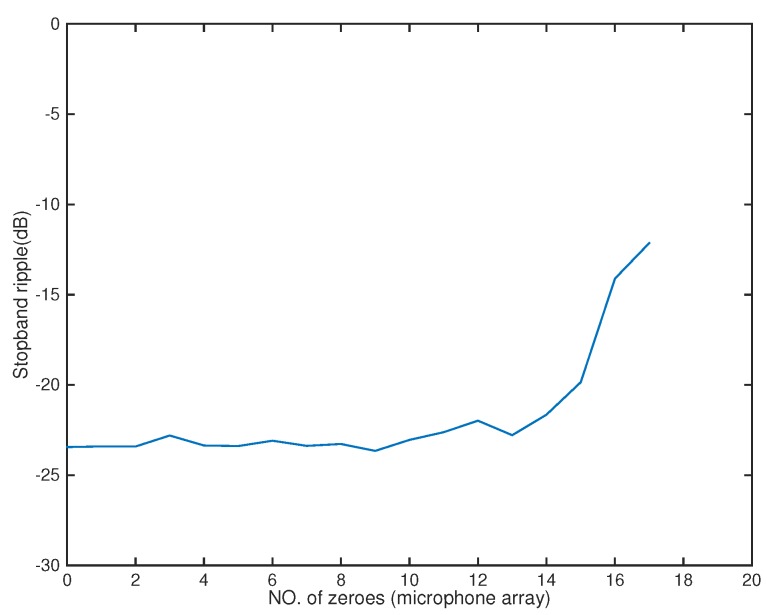
Stopband ripple for microphone arrays (Ex2).

**Figure 11:**
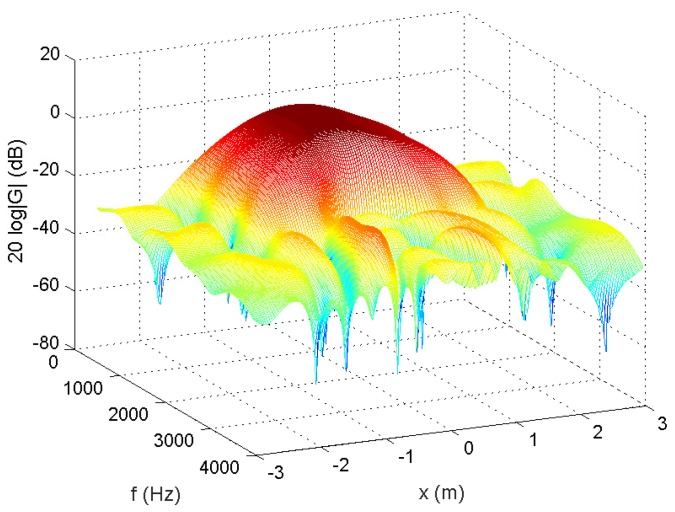
Amplitude of G(r,f), 41.67% of zeroes for filters (Ex2).

**Figure 12:**
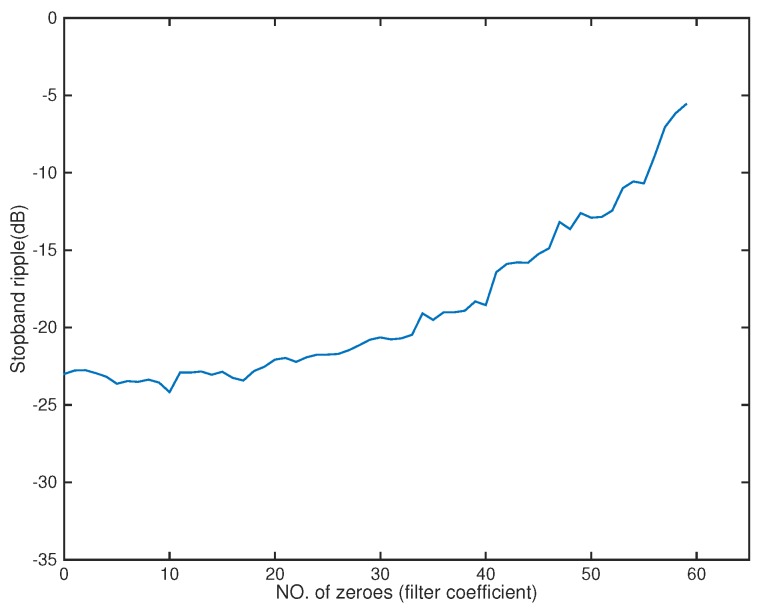
Stopband ripple for filters (Ex2).

A 12-tap FIR filter behind each element was used. The number of microphones could be decreased to 5 (Figure 8). The amplitude of the actual response G(r,f) is shown in Figure 9. From Figure 10, we can see that the designed beamformer array could be simplified significantly without greatly affecting the performance. After pruning the microphone array, we could then carry out the sparse filter design problem. The amplitude of the actual response G(r,f) is shown in Figure 11. It is observed in Figure 12 that the performance was not affected greatly, even when the sparseness increased to about 41% of zero elements for the filter coefficient.

## 5. Conclusions

In this paper, we studied the sparse beamformer design problem for both array and filters. A novel array pruning problem was formulated and an optimization algorithm was proposed to prune the microphones sequentially. In finding sparse beamformer filters, a perturbation exchange algorithm was proposed to solve the sparse design problem with the successful thinning technique. We studied the performance of the optimized designs with several examples. We demonstrated that there was a region of approximately equal performance for both array pruning and sparse filter design. As a future extension, it would be of interest to study the convergence properties of the overall optimization algorithm.

## References

[1] Brandstein M., Ward D. (2001). Microphone Arrays: Signal Processing Techniques and Applications.

[2] Kellermann W., Havelock D., Kuwano S., Vorlander M. (2008). Beamforming for speech and audio signals. Handbook of Signal Processing in Acoustics.

[3] Ryan J., Goubran R. (2000). Array optimization applied in the near field of a microphone array. IEEE Trans. Speech Audio Process..

[4] Kennedy R., Ward D., Abhayapala T. (1999). Nearfield beamforming using radial reciprocity. IEEE Trans. Signal Process..

[5] Lim Y.C., Lian Y. (1993). The optimum design of one and two-dimensional FIR filters using the frequency response masking technique. IEEE Trans. Circuits Syst. II.

[6] Lau B.K., Leung Y.H., Teo K.L., Sreeram V. (1999). Minimax filters for microphone arrays. IEEE Trans. Circuits Syst. II.

[7] Chan S.C., Chen H.H. (2007). Uniform concentric circular arrays with frequency-invariant characteristics-theory, design, adaptive beamforming and DOA estimation. IEEE Trans. Signal Process..

[8] Nordholm S., Rehbock V., Teo K.L., Nordebo S. (1998). Chebyshev approximation for the design of broadband beamformers in the near field. IEEE Trans. Circuits Syst. II.

[9] Yiu K.F.C., Grbic N., Teo K.L., Nordholm S. (2002). A new design method for broadband microphone arrays for speech input in automobiles. IEEE Signal Proc. Lett..

[10] Yiu K.F.C., Yang X.Q., Nordholm S., Teo K.L. (2003). Near-field broadband beamformer design via multidimensional semi-infinite linear programming techniques. IEEE Trans. Speech Audio Process..

[11] Feng Z.G., Yiu K.F.C., Nordholm S. (2011). A two-stage method for the design of near-field broadband beamformer. IEEE Trans. Signal Process..

[12] Feng Z.G., Yiu K.F.C. (2014). The design of multi-dimensional acoustic beamformers via window functions. Digit. Signal Process..

[13] Feng Z.G., Yiu K.F.C., Nordholm S. (2015). Performance limit of broadband beamformer designs in space and frequency. J. Optim. Theory Appl..

[14] Baran T., Wei D., Oppenheim A.V. (2010). Linear programming algorithms for sparse filter design. IEEE Trans. Signal Process..

[15] Jiang A., Kwan H.K., Lim Y., Kwan H.K., Siu W.C. (2016). Recent advances in sparse FIR filter design using *l*_0_ and *l*_1_ optimization techniques. Trends in Digital Signal Processing.

[16] Zhang L.P., Wu S.Y., Lopez M.A. (2010). Optimizing floating point units in hybrid FPGAs. SIAM J. Optim..

[17] Yiu K.F.C., Gao M.J., Feng Z.G. (2012). Design of near-field broadband beamformer using semi-definite programming. Int. J. Innov. Comput. Inf. Control.

[18] Oliveri G., Donelli M., Massa A. (2009). Linear array thinning exploiting almost difference sets. IEEE Trans. Antennas Propag..

[19] Fernandez-Delgado M., Rodriguez-Gonzalez J., Iglesias R., Barro S., Ares-Pena A. (2010). Fast array thinning using global optimization methods. J. Electromagn. Waves Appl..

[20] Feng Z.G., Yiu K.F.C., Nordholm S. (2012). Placement design of microphone arrays in near-field broadband beamformers. IEEE Trans. Signal Process..

[21] Li Z.B., Yiu K.F.C., Feng Z.G. (2013). A hybrid descent method with genetic algorithm for microphone array placement design. Appl. Soft Comput..

[22] Honig M., Madhow U., Verdu S. (1995). Blind adaptive multiuser detection. IEEE Trans. Inf. Theory.

[23] Darsena D., Verde F. (2007). Minimum-mean-output-energy blind adaptive channel shortening for multicarrier simo transceivers. IEEE Trans. Signal Process..

[24] Gao M.J., Yiu K.F.C., Wu S.Y. (2018). A perturbation exchange algorithm for convex semi-infinite programming with applications in sparse beamformer. Pac. J. Optim..

